# Kojic Acid Production from Agro-Industrial By-Products Using Fungi

**DOI:** 10.1155/2014/642385

**Published:** 2014-03-23

**Authors:** Ismael A. El-Kady, Abdel Naser A. Zohri, Shimaa R. Hamed

**Affiliations:** ^1^Botany Department, Faculty of Science, Assiut University, Assiut 71515, Egypt; ^2^Microbial Biotechnology Department, National Research Center, Dokki 12622, Egypt

## Abstract

A total of 278 different isolates of filamentous fungi were screened using synthetic medium for respective ability to produce kojic acid. Nineteen, six, and five isolates proved to be low, moderate, and high kojic acid producers, respectively. Levels of kojic acid produced were generally increased when shaking cultivation was used rather than those obtained using static cultivation. A trial for the utilization of 15 agro-industrial wastes or by-products for kojic acid production by the five selected higher kojic acid producer isolates was made. The best by-product medium recorded was molasses for kojic acid. * A. flavus* numbers 7 and 24 were able to grow and produce kojic acid on only 12 out of 15 wastes or by-products media. The best medium used for kojic acid production by * A. flavus* number 7 was rice fragments followed by molasses, while the best medium used for kojic acid production by * A. flavus* number 24 was the molasses followed by orange, pea, and rice fragments. An attempt for production of kojic acid using a 1.5 L laboratory fermentor has been made. * Aspergillus flavus* number 7 was used and grown on molasses medium; maximum level (53.5 g/L) of kojic acid was obtained after eight days of incubation.

## 1. Introduction

Kojic acid is a metabolic product of several species of the economically valuable genus* Aspergillus*. This mold is used in the production of a number of foods, including* miso* (soybean paste),* shoyu *(soy sauce), and* sake*, which are produced throughout the world. This mold is also used in the production of other fermented products including* amazake* (a sweet beverage),* shochu *(a distilled liquor), and* mirin *(a sweet, alcoholic seasoning), which are consumed primarily among the Japanese. Because kojic acid is produced during the fermentation of these historically used dietary staples, it has a long history of consumption. Health foods containing kojic acid are widely sold in Japan [1]. Kojic acid is permitted for addition to foods in Japan [2]. Kojic acid has been added to food as an antioxidant [3], as a preservative to prevent formation of warmed-over flavor in beef [4], as a processing aid to inhibit the formation of nitrosopyrrolidine in fried bacon [5], and to produce reddening in unripe strawberries [6]. It has also been used as a starting material for synthesis of the flavor enhancer's maltol [7]. Yellow product formation takes place when both kojic acid and* o*-quinones are present. Kojic acid and some of its derivatives are used in cosmetic preparations to achieve a skin-lightening effect by inhibiting melanin formation and through a UV light protective action. Kojic acid also enhances shelf life of the product through its preservative actions against both chemical and microbial degradation [8, 9]. In addition, kojic acid has been used as an antibiotic, pesticide, and analytical chemical (in the determination of thorium and rare earths) as recorded by many investigator [10–12]. Utilization of industrial waste or by-products for the fungal production of useful products has been recommended by many investigations such as glycerol production by filamentous fungi using cheese whey [13–15], lipid, and sterol and ergosterol production by fungi using sugar cane molasses or cheese whey [16–18] and side-chain degradation and some biological transformation of progesterone by fungi using sugar cane molasses [19] and cyclosporin A production by fungi grown on agro-industrial wastes of some fruits, vegetables, and pickles as well as molasses and corn steeps [20–22]. The objective of this study was to study the following: the potentialities of kojic acid and production by 278 different fungal isolates; comparison between static and shaking cultivation methods for the production; optimization of both nutritional and environmental factors affecting the acid production; and utilization of 15 kinds of agro-industrial wastes or by-products by the high kojic acid producers for acid formation as well as production of this product on semi-industrial scale using a laboratory fermentor.

## 2. Materials and Methods

### 2.1. Tested Isolates

Two hundred and seventy-nine isolates of seventy-three species and one species variety represented sixteen genera of filamentous fungi collected during this study were examined for kojic acid production. These different isolates were obtained from the Botany Department, Faculty of science, Assiut University, Egypt, and AUMC (Assiut University Mycological Center), Assiut University.

### 2.2. Medium and Fermentation

The optimized medium for kojic acid production by* A. flavus* Link as proposed by Madiha et al. [23] was used in all experiments for inoculum preparation and also for kojic acid fermentation. The experimental cultures were grown in 250 mL Erlenmeyer flasks, each containing 50 mL of the synthetic medium. The flasks were sterilized at 121°C for 20 min and inoculated after cooling with 2 mL of 7- to 10-day-old cultures. The inoculum spore suspensions were prepared by adding sterile distilled water to the slant culture, followed by gentle agitation. The final concentration of spore's suspension was about 5 × 10^6^ spores per mL. The cultures were incubated at 28 ± 2°C as stationary cultivation for 15 days.

### 2.3. Agro-Industrial Wastes and By-Products Used as Culture Media

*(1) Fruits, Vegetables, and Pickle Wastes and Agriculture By-Products*. Each individual fungal isolate of the highly kojic acid producers (five isolates) was cultivated on semisynthetic culture media. Each medium contained 100 g of agro-industrial waste product, individually, of each of orange, peach, apple, and apricot as fruit wastes; pea, mixed vegetable, and kidney beans as vegetables wastes; carrot and turnip as pickle wastes as well as wheat bran, rice fragments, and rice husk as agriculture by-products added to one liter of distilled water and supplemented with 5 g/L of yeast extract. All industrial wastes were collected from different juices, vegetables canning, and pickles factories located at the industrial areas of different governorates in Egypt, while the three agricultural by-products were collected from different farms of different governorates in Egypt.

*(2) Corn Steep Liquor*. Corn steeps liquor was prepared by using sweet corn maize. 100 g from the substrate was put in 2000 mL Erlenmeyer flasks and completed to 2000 mL by distilled water and cooked on a very quiet flame for 12 h; after that, these were filtered through a muslin cloth and used. Each individual fungal isolate of the highly kojic acid producers was cultivated on a medium consisting of 100 mL of corn steep liquor added to 900 mL of distilled water to complete one liter medium.

*(3) Cheese Whey*. Salted cheese whey is a by-product formed from milk during the production of cheese (both soft and hard cheese). Whey used during this work was produced from milk composed of 1 : 1 cow's and buffalo's milk which were used for production of white soft (Domiati type) cheese. Whey sample was kindly provided by Dairy Department, Faculty of Agricultural, Assiut University. Samples of whey (8% NaCl) were centrifuged (5000 rpm, 10 min), the sediment was discarded, and samples of supernatant were used as it is.

*(4) Black-Strap Molasses*. Black-strap molasses supplemented from El-Hawamdya sugar cane factory were tested as natural medium for cultivation of the experimental organisms. The molasses sample was centrifuged (5000 rpm, 10 min), the muddy sediment was discarded, and samples of supernatant were tested. Each individual fungal isolate of the highly kojic acid producers was cultivated on liquid semisynthetic medium of the following composition: supernatant molasses sample, 100 mL; yeast extract, 5.0 g; and completed to one liter distilled water. The pH of the different media was adjusted at 3.0 before sterilization. The cultures were incubated at 28 ± 2°C on rotary shaker (220 rpm) for 10 days.

### 2.4. Quantitative Determination of Kojic Acid

Kojic acid was determined using a spectrophotometric method with 2,6 dichlorophenolindophenol (DCIP) as recorded by Tanigaki et al. [7].

### 2.5. Production of Kojic Acid on Semi-Industrial Scale

A 1.5-liter B. Braun stirred tank (Biostat. A) fermentor (from B. Braun Biotech. International, Sortorius group, GmbH, Schwarzenberger, Germany) with one liter working volume was used in this study. The fermentor was equipped with pH, temperature, agitation, dissolved oxygen tension (DOT), and foam controllers. Seed cultures were carried out in 250 mL flask containing 50 mL of medium, held on a rotary shaker at 150 rpm, at 28°C for 48 h. Seed culture flask (50 mL) from fungal isolates (*Aspergillus flavus* number 7), which proved to be the higher kojic acid producer, was used to inoculate the fermentor at 30°C. Fermentation lasted around 14 days. The culture medium was modified synthetic medium consisting of (g/L): glucose, 100; yeast extract, 5.0; KH_2_PO_4_, 1.5; and MgSO_4_·7H_2_O, 0.5. The pH was adjusted to 3.0, temperature at 30°C, and agitation at 400 rpm, while the DOT in the culture broth was controlled via a sequential cascade control as air flow rate. The maximum and minimum set points of permitted airflow rates were 1.2 L/min and 0.1 L/min, respectively. The DOT during fermentation was controlled at medium (~50%) of saturation.

## 3. Results and Discussion

Screening the abilities of 278 different fungal isolates belonging to 16 genera and 71 species in addition to one species variety for kojic acid production was an aim in this study.* Aspergillus *was represented by 135 isolates of 18 species and one variety belonging to nine sections (Table 1). High concentrations (more than 15 g/L medium) of kojic acid were produced by only three isolates of* A. flavus* (numbers 7, 23, and 24) and two isolates of* A. flavus* var.* columnaris* (numbers 36 and 41). Moderate levels (5 to 15 g/L medium) were obtained by five* Aspergillus* isolates (one of each of* A. flavus* var.* columnaris*, number 39;* A. terreus*, number 131, and* A. versicolor*, number 143 in addition to two isolates of* A. tamarii* (numbers 129 and 130), while low concentrations (less than 5 g/L medium) were obtained by eight* Aspergillus* isolates as follows: three isolates of* A. flavus* (numbers 4, 9, and 32), two of* A. phoenicis* (numbers 119 and 120) in addition to one isolate of each of* A. sclerotiorum* (number 121),* A. flavus* var.* columnaris* (number 43), and* A. wentii *(number 145). It is worth mentioning that 12 out of the 18* Aspergillus* isolates, which recorded as kojic acid producers, belonging to two species (*A. flavus* and* A. tamarii*) and one species variety (*A. flavus* var.* columnaris*) of section flavi (Table 1). Also, one isolate of each of* Emericella nidulans* (number 148) and* Eurotium amstelodami *(number 155) as species belonging to* Aspergillus* related genera (based on anamorph/teleomorph) had the ability to produce low levels of kojic acid. Kharchenko [24] studied the ability of 98 strains of* A. flavus* to form kojic acid and recorded 14 strains of them as highly active. This is nearly similar to those recorded in the present investigation (three out of 29 isolates tested of* A. flavus *were recorded as highly producers). Previously, several species of* Aspergillus *were recorded as kojic acid producers such as* A. flavus* [25–31],* A. oryzae* [32, 33],* A. fumigatus* [28, 34, 35],* A. candidus*, [33, 36],* A. awamori, A. clavatus., A. ustus,* and* A. wentii* [33]. Also, Manabe et al. [33] recorded* A. nidulans* (=anamorph of* Emericella nidulans*) as kojic acid producer. Parrish et al. [37], examined the production of kojic acid by 14 species of* Aspergillus* and recorded the production of the acid by each of* A. clavatus*,* A. flavus*,* A. fumigates*,* A.oryzae*,* A. parasiticus*,* A. tamarii*,* A. ustus*, and* A. nidulans* (=*Emericella nidulans*). Production of kojic acid by* A. flavus* var.* columnaris*,* A. terreus*,* A. versicolor*,* A. phoenicis*,* A. sclerotiorum*, and* Eurotium amstelodami* recorded in this study for first time, according to the available literatures.

Seventy-six isolates of 24 species of* Penicillium* belonging to four subgenera were tested for respective abilities to produce kojic acid (Table 2). Only one isolate of each of* P. spinulosum* (number 230),* P. janthinellum* (number 216),* P. aurantiogriseum *(number 154),* P. frequentans *(number 211), and* P. godlewski* (number 214) had the ability to produce kojic acid at low concentrations (less than 5 g/L medium). Ariff et al. [31] and Burdock et al. [38] reported that kojic acid could be produced by many species of* Aspergillus* and* Penicillium.* Parrish et al. [37] tested eight species of* Penicillium *for kojic acid production and found that each of* P. puberulum*,* P. estmogenum*,* P. albidum,* and* P. daleae* had the ability to produce kojic acid. Production of kojic acid by* P. citrinum*,* P. griseofulvum*,* P. rubrum*, and* P. purpurogenum* was previously recorded by Manabe et al. [33].

From 60 isolates belonging to 27 species of 12 genera representing Hyphomycetes (22 species of 9 genera) and Zygomycetes (five species of three genera) were tested for kojic acid production. Only one isolate of each of* Fusarium equiseti* number 271 (teleomorph:* Gibberella intricans*),* F. moniliforme* number 283 (teleomorph:* G. fujikuroi*),* F. oxysporum* number 287,* F. tricinctum* number 299, and* Chaetomium globosum* number 242 proved to be producers of kojic acid at low or moderate levels (Table 3). All these producers belonged to Hyphomycetes while all the tested isolates of Zygomycetes completely failed to produce any detectable amounts of kojic acid. According to the available literatures, there is no record on the production of kojic acid by any members of Hyphomycetes or Zygomycetes. The higher producer isolates of kojic acid (*A. flavus* numbers 7, 23, and 24 and* A. flavus* var.* columnaris* numbers 36 and 41) were selected for comparison between static and shaking cultivation methods. Generally, the concentrations of kojic acid produced were increased when submerged cultivation (shaking) was used than those recorded using surface cultivation (static) (Table 4). Kojic acid levels produced by the five isolates grown using shaking cultivation were fluctuated between 18.3 and 34.4 g/L medium, while those levels recorded using static cultivation ranged from 16.3 to 21.4 g/L medium. The high concentration of kojic acid (34.4 g/L) was formed by* A. flavus* number 7 using shaking cultivation. Nearly similar results were recorded by Ariff et al. [31]. They found that the level of kojic acid accumulated by* A. flavus* strain 44-1 using rotary shaker was 32.5 g/L. Rosfarizan and Ariff [39] found that the highest level of kojic acid production by* A. flavus* strain 44-1 reached 39.9 g/L in submerged batch fermentation.

Manabe et al. [32] produced kojic acid at 40 mg/mL medium (=40 g/L)* A. flavus* isolated from Japanese fermented foods. El-Kady et al. [35] recorded 57–59 mg of kojic acid per mL medium formed by* A. fumigatus* isolated from Buffalo pneumonia. High concentration of kojic acid (60 g/L medium) was recorded by El-Sharkawy [30] using calcium alginate immobilization technique for kojic acid production by* A. flavus* ATCC 9179. Kwak and Rhee [40] produced kojic acid using, also, immobilized cells of* A. oryzae* and recorded a very high kojic acid production level (reached up to 80 g/L) in repeated batch culture. Higher final concentrations of kojic acid in solution caused kojic acid to crystallize in the form of fine needles [9, 40] and this is very useful for easy and low cost recovery. On the other hand, low level of kojic acid was recorded by Ogawa et al. [41], who reported that the maximum yield of kojic acid was around 20 mg/mL formed by* A. oryzae* NRRL 484 using shaking culture. Wakisaka et al. [42] found that the kojic acid level produced by* A. oryzae* NRRL 484 the same isolate used by Ogawa et al. [41] using shaking flask cultures was 24 g/L. The superior isolate (*A. flavus* Number 7) for kojic acid production (which formed 34.4 g/L of kojic acid using shaking cultivation) was selected, using this cultivation method for a series of experiments to determine the effect of some nutritional and environmental conditions on the efficiency of kojic acid production by this isolate. This is for maximization of kojic acid production. This study explained that optimal nutritional conditions for this isolate were 100 g/L glucose, 5.0 g/L yeast extract, and 1.5 g/L KH_2_PO_4_ as carbon, nitrogen, and phosphorus sources, respectively. The optimal pH, temperature, and incubation period as environmental conditions were pH 3, 30°C, and 10 days, respectively. These results are completely similar to those recorded by several investigators [31, 39, 43, 44]. An attempt has been made, in this study, to investigate the possibility to utilization of agro-industrial wastes or by-products as natural medium for kojic acid production by the five high producer isolates (*A. flavus* numbers 7, 23, and 24 and* A. flavus* var.* columnaris* numbers 36 and 41). The agro-industrial wastes and by-products used in this study were pea, kidney bean, and mixed vegetables wastes; the wastes of juice production of each of apple, apricot, orange, and peach; the wastes of other vegetables used as pickles, namely, carrot and turnip; three industrial by-products, namely, corn steep liquor, molasses, and cheese whey; in addition to three agricultural by-products as wheat bran, rice husk, and rice fragments (Tables 5, 6, and 7). Generally, kojic acid production levels by the five tested fungal isolates grown on any wastes or by-products under investigation were relatively low (ranged from 0.0 to 21.2 g/L medium) comparing to those levels produced by the same fungal isolates on synthetic medium which ranged from 18.3 to 34.4 g/L medium. Low levels of kojic acid production by the high producer isolates grew on a medium containing carbon sources other than glucose were previously recorded [39, 44, 45]. Rosfarizan and Ariff [39] reported that the level of kojic acid production by* A. flavus* strain 44-1 was 4.4 g/L in submerged batch fermentation using lactose as carbon source. Also, they reported that glucose was the best out of seven carbon sources tested (glucose, xylose, sucrose, fructose, lactose, maltose, and starch) for kojic acid production. Rosfarizan et al. [44] found that the maximum yield of kojic acid by* A. flavus* strain 44-1 grown on gelatinized sago starch as carbon source was 4.51 g/L. Moreover, no kojic acid was produced by* A. oryzae* when starch was used as carbon source as recorded by Kitada et al. [43]. Rice fragments and molasses as by-products were relatively suitable substrates, for kojic acid production by the five fungal isolates tested. The two tested isolates of* A. flavus* var.* columnaris *(numbers 36 and 41) in addition to one isolate of* A. flavus* (number 7) could use rice fragments as by-product medium and produce relatively high levels of kojic acid (21.2, 18.2 and 12.1 g/L, resp.), while the other two isolates tested of* A. flavus* (numbers 23 and 24) formed relatively high levels of the acid (9.3 and 5.1 g/L, resp.) on molasses medium (Table 7).

Egyptian sugar cane molasses contain about 44% as total sugar (glucose, sucrose, and fructose), 0.46% as total nitrogen in addition to detectable amounts of some vitamins such as riboflavin and thiamin [46, 47]. Lai et al. [48] reported that the main chemical characteristics of rice husk contain: carbon (45.3%), hydrogen (5.5%), nitrogen (0.67%), sulfur (0.29%), and chlorine (0.29) in addition to detectable amounts of potassium (1630 ppm), calcium (94 ppm), iron (202 ppm), sodium (207 ppm), zinc (24 ppm), magnesium (699), phosphorus (94 ppm), and other. Presence of these compounds in each of molasses and rice husk may favor kojic acid production. El-Refai and El-kady [49] and Ghanem et al. [50] reported the possible utilization of molasses for sterols production by yeast and filamentous fungi, respectively. Kahraman and Yesilada [51] used industrial and agricultural wastes as substrates for laccase production by* Coriolus versicolor* ATCC 200801 and* Funalia trogii* ATCC 200800 as white rot fungi and recommended using these waste in the production of important lignocellulolytic and other biotechnological enzymes, respectively. Sallam et al. [52] used a medium composed of cane sugar molasses (3%) and corn steep liquor (1%) for cyclosporin A (which used as a powerful immunosuppressant to prevent graft rejection in transplantation surgery) production by* A. terreus* and recorded the production of 45.23 mg cyclosporin A per each one liter medium. More recently, Ragaa Kotby [20] found that ten fungal isolates (one of each of* A. ustus, Fusarium nivale, F. oxysporum, F. moniliforme, Trichoderma hamatum, *and* T. pseudokoningii* in addition to four isolates of* T. harzianum*) had the ability to grow well and produce cyclosporin A at levels fluctuated between 400 and 1200 *μ*g/50 mL of 10% molasses medium.

Production of kojic acid by the superior producer isolate (*A. flavus* number 7), recorded in this study, grew on the synthetic medium using a 1.5 laboratory fermentor (semi-industrial scale) was the target in the last experiment of this part in this investigation (Table 8). The results revealed that at the first two days, kojic acid concentration reached 28.6 g/L. After this time, kojic acid levels were increased gradually with the increase of fermentation time and reaching maximum level (53.5 g/L) after eight days of inoculation. Gradual decrease in kojic acid concentrations was recorded with further extension of fermentation period. These results are in harmony with that previously obtained by Ariff et al. [31]. They used a fermentor (2 L B, Braum stirred tank fermentor, Biostat. B) For kojic acid production by* A. flavus* strain 44-1 and found that kojic acid concentration reached up to 36.5 g/L after 11 days. Also, reduction of kojic acid formation after the concentration became maximum was observed by Ariff et al. [31] and El-Assar [53]. They attribute this reduction to degradation of kojic acid to oxalic and acetic acid by the mycelium under glucose depleted conditions [23, 31, 54, 55]. Futamura et al. [56] produced kojic acid by* A. oryzae* MK-107-39 in a jar fermentor and recorded that level of kojic acid reached up to 40 g/L. On the other hand, Wakisaka et al. [42] produced kojic acid by* A. oryzae* NRRL 484 using continuous membrane surface liquid culture technique and recorded that kojic acid concentration reached 45 mg/mL medium and nearly constant after 15 days of cultivation for over 70 days.

In view of the wide use of kojic acid as a food ingredient (flavor enhancers, antioxidant, and/or discoloration) [57, 58], skin-lightening agent in cosmetic or dermatological preparations [22, 59], bacterial inhibitor, pain Killer, and anti-inflammatory agent in medical field [60], preventer for the undesirable melanosis (blackening) of agricultural products [45, 61] and many other uses, it seemed necessary to conduct a thorough investigation of production of kojic acid on large scale.

## Figures and Tables

**Table 1:** Production of kojic acid by different isolates belonging to various species and varieties of *Aspergillus* and their teleomorph.

Organisms	Code number	Total isolates tested	−ve isolates	+ve isolates		
				Low*	Moderate**	High***
Subgenus: *circumdati *						
Section: *candidi *						
*A. Candidus* Link	2	1	1	—	—	—
Section: *circumdati *						
*A. melleus* Yukowa	78–80	3	3	—	—	—
*A. Ochraceus* Wilhelm	100–113	14	14	—	—	—
*A. sclerotiorum* Hoper	121	1	—	1	—	—
*A. Sulphureus* (Fres.) Thom and Church	122, 123	2	2	—	—	—
Section: Flavi						
*A. flavus* Link	4–32	29	23	3	—	3
*A. flavus* var. *columnaris* Raper and Fennell	36–63	28	25	1	1	2
*A. Oryzae* (Ahlburg) Cohn	115, 116	2	2	—	—	—
*A. Parasiticus* Spear	117, 118	2	2	—	—	—
*A. tamarii* Kita	129, 130	2	—	—	2	—
Section: Nigri						
*A. aculeatus* Lizuka	1	1	1	—	—	—
*A. niger* van Tieghem	81–99	19	19	—	—	—
*A. phoenicis* (Cda.) Thom	119, 120	2	—	2	—	—
Section: *wentii *						
*A. wentii* Wehmer	144–146	3	2	1	—	—
Subgenus: *fumigati *						
Section: Fumigati						
*A. fumigatus* Fresenius	64–75	12	12	—	—	—
Subgenus: Nidulantes						
Section: Flavipedes						
*A. flavipes* (Bain. and Sart) Thom and Church	3	1	1	—	—	—
Section: Terii						
*A. terreus* Thom	131–139	9	8	—	1	—
Section: Versicolores						
*A. Janus* Raper and Thom	77	1	1	—	—	—
*A. sydowii* (Bain. and Sart) Thom and Church	124–127	4	4	—	—	—
*A. versicolor* (Vuill.) Tiraboschi	143	1	—	—	1	—

*Emericella nidulans* (Eidam) Vuillemin	147–150	4	3	1	—	—
*Eurotium amstelodami* Mangin	151	1	—	1	—	5

Total	—	142	122	10	5	5

*Less than 5 g/L medium kojic acid.

**5–15 g/L medium kojic acid.

***More than 5 g/L medium kojic acid.

**Table 2:** Production of kojic acid by different isolates belonging to various species of *Penicillium*.

Organisms	Code number	Total isolates tested	−ve isolates	+ve isolates		
				Low*	Moderate**	High***
Subgenus: *aspergilloides *						
*P. Capsulatum *Raper and Fennell	159-163	5	5	—	—	—
*P. lividum *Westling	218–220	3	3	—	—	—
*P. spinulosum *Thom	230, 231	2	1	1	—	—
Subgenus: *biverticillium *						
*P. funiculosum* Thom	213	1	1	—	—	—
*P. purpurogenum* Stoll	227, 228	2	2	—	—	—
*P. rugulosum* Thom	229	1	1	—	—	—
Subgenus: furactum						
*P. citrinum *Thom	187–201	15	15	—	—	—
*P. corylophilum* Dierckx	202, 203	2	2	—	—	—
*P. herquei* Bain. and Sart.	215	1	1	—	—	—
*P. janthinellum* Biourge	216, 217	2	1	1	—	—
Subgenus: *penicillium *						
*P. albidum *Sopp	152	1	1	—	—	—
*P. atramentosum *Thom	153	1	1	—	—	—
*P. aurantiogriseum* Dierckx	154	1	—	1	—	—
*P. camemberti* Thom	156, 157	2	2	—	—	—
*P. chrysogenum* Thom	164–185	22	22	—	—	—
*P. cyaneofulvum* Biourge	205	1	1	—	—	—
*P. cyclopium* Westling	206	1	1	—	—	—
*P. digitatum* (Pers. ex Fr.) Saccardo	207	1	1	—	—	—
*P. expansum *Link ex Gray	210	1	1	—	—	—
*P. frequentans* Westling	211, 212	2	1	1	—	—
*P. godlewski* Zaleski	214	1	—	1	—	—
*P. nigricans *(Bain.) Thom	222–226	5	5	—	—	—
*P. somniferum * Thom	234	2	2	—	—	—
*P. viridicatum* Westling	236	1	1	—	—	—

Total	—	76	71	5	—	—

*Less than 5 g/L medium kojic acid.

**5–15 g/L medium kojic acid.

***More than 5 g/L medium kojic acid.

**Table 3:** Production of kojic acid by different isolates belonging to Hyphomycetes and Zygomycetes.

Organisms	Code number	Total isolates tested	−ve isolates	+ve isolates		
				Low*	Moderate**	High***
Group: Hyphomycetes						
Family: Dematiaceae						
*Alternaria alternata* (Fries.) Keister	239–241	3	3	—	—	—
*Chaetomium globosum* Kunze	242	1	—	1	—	—
*Pleospora herbarum* (Fr.) Robenh. Exces. and De Notaris	247	1	1	—	—	—
*Scopulariopsis brevicaulis* (Saccardo) Bainier	248	1	1	—	—	—
*Stachybotrys chartarum* (Ehrenberg) Hughes	250, 251	2	2	—	—	—
*Stachybotrys theobromae* Hansf.	252	1	1	—	—	—
*Torula herbarum* (Pres.) Link	254	1	1	—	—	—
*Trichoderma hamatum* (Bon.) Bain.	255	1	1	—	—	—
*T. koningii* Oudemans	258	1	1	—	—	—
*T. longibrachiatum* Rifai	259	1	1	—	—	—
*T. polysporum* (Link ex pres.) Rifai	262, 263	2	2	—	—	—
Family: Moniliaceae						
*Acremonium strictum* W. Gams	237	1	—	—	—	—
Family: Tuberculariaceae						
*Fusarium aquaeductuum* (Radlk. and Rabenh.)	266	1	1	—	—	—
*F. chlamydosporum* Wollenw. and Reinking	267–269	3	3	—	—	—
*F. equiseti* (Corda) Saccardo	270, 271	2	1	1	—	—
*F. lateritium* Nees and Sys.	272	1	1	—	—	—
*F. moniliforme* Sheldon	273–283	11	10	1	—	—
*F. oxysporum* Schlecht	284–295	12	11	—	1	—
*F. proliferatum* (Matsushima) Nirenberg	296	1	1	—	—	—
*F. solani* (Mart.) Saccardo	297	1	1	—	—	—
*F. subglutinans* (Wollenw. and Reinking) Nelson	298	1	1	—	—	—
				
*F. tricinctum* (Corda) Saccardo	299	1	—	—	—	—
Group: Zygomycetes						
Family: Mucoraceae						
*Cunninghamella echinulata* (Thaxter) Thaxt. ex Blakasles	315	1	1	1	—	—
*C. elegans* Landner	316, 317	2	2	—	—	—
*Mucor Circinelliodes* van Tieghem	319–321	3	3	—	—	—
*M. fuscus* Bainier	329, 331, 332	3	3	—	—	—
Family: Syncephalastraceae						
*Syncephalastrum racemosum* Cohn and Schroter	343	1	1	—	—	—

Total	—	60	55	4	1	—

*Less than 5 g/L medium kojic acid.

**5–15 g/L medium kojic acid.

***More than 5 g/L medium kojic acid.

**Table 4:** Comparison between surface (static) and submerged (shaking) cultivation for kojic acid production (g/L) using the synthetic medium by the five highly producer organisms.

Organisms	Code number	Static cultivation	Shaking cultivation
*Aspergillus flavus *	24	16.3	18.3
*A. flavus *	23	18.5	28.5
*A. flavus *	7	21.4	34.4
*A. flavus * var. *columnaris *	36	21.4	22.8
*A. flavus *var.* columnaris *	41	15.3	26.3

**Table 5:** Production of kojic acid (g/L) by the selected five highly producer organisms grown on vegetables and pickles wastes as well as synthetic media for 10 days as shaking cultivation.

Fungal isolates tested	Code number	Synthetic medium	Kind of vegetable wastes			Kind of pickles wastes	
			Pea	Kidney bean	Mixed vegetable	Carrot	Turnip
Control*	—	—	—	—	—	—	—
*Aspergillus flavus *	24	18.3	5.5	0.4	0.8	0.0	1.5
*A. flavus *	23	28.5	2.1	0.1	0.3	0.6	0.5
*A. flavus *	7	34.4	2.5	0.8	0.2	0.0	0.6
*A. flavus *var. *columnaris *	36	22.8	1.1	2.0	0.2	0.0	0.9
*A. flavus *var. *columnaris *	41	26.3	0.9	0.1	1.0	0.0	1.6

*Control: wastes or by-products without fungal inoculum.

**Table 6:** Production of kojic acid (g/L) by the selected five highly producer organisms grown on fruit wastes as well as synthetic media for 10 days as shaking cultivation.

Fungal isolates tested	Code number	Synthetic medium	Kind of fruit wastes			
			Apple	Apricot	Orange	Peach
Control*	—	—	—	—	—	—
*Aspergillus flavus *	24	18.3	2.6	3.1	5.9	2.7
*A. flavus *	23	28.5	4.2	1.5	4.1	0.5
*A. flavus *	7	34.4	2.2	1.2	2.0	1.0
*A. flavus *var. *columnaris *	36	22.8	0.9	0.4	1.3	0.6
*A. flavus *var. *columnaris *	41	26.3	2.1	0.6	0.8	0.7

*Control: wastes or by-products without fungal inoculum.

**Table 7:** Production of kojic acid (g/L) by the selected five highly producer organisms grown on agriculture and industrial by-products as well as synthetic media for 10 days as shaking cultivation.

Fungal isolates tested	Code number	Synthetic medium	Kind of agriculture by-products			Kind of industrial by-products		
			Wheat bran	Rice fragment	Rice husk	Corn steep liquor	Molasses	Whey
Control*	—	—	—	—	—	—	—	—
*Aspergillus flavus *	24	18.3	3.0	5.3	2.8	0.0	9.3	0.0
*A. flavus *	23	28.5	1.0	0.9	0.1	1.3	5.1	0.1
*A. flavus *	7	34.4	0.7	12.1	0.1	0.0	6.2	0.0
*A. flavus *var. *columnaris *	36	22.8	2.0	21.2	0.1	3.1	4.0	0.0
*A. flavus *var. *columnaris *	41	26.3	1.6	18.2	1.6	0.4	3.7	0.0

*Control: wastes or by-products without fungal inoculum.

**Table 8:** Production of kojic acid (g/L) by the selected five highly producer isolate (*A. flavus* number 7) grown on the synthetic medium for 14 days using a laboratory fermentor.

Fermentation period (days)	Kojic acid (g/L)
2	28.6
4	31.5
6	48.3
8	53.5
10	51.6
12	50.8
14	47.1
